# MOSFET dosimeter characterization in MR‐guided radiation therapy (MRgRT) Linac

**DOI:** 10.1002/acm2.12799

**Published:** 2019-12-18

**Authors:** Poonam Yadav, Abdelbasset Hallil, Dinesh Tewatia, David A. P. Dunkerley, Bhudatt Paliwal

**Affiliations:** ^1^ Department of Human Oncology University of Wisconsin‐Madison Madison WI USA; ^2^ Best Medical Canada Ottawa ON Canada

**Keywords:** beam QA, dosimetry, IMRT, low magnetic field, MOSFET, MRgRT

## Abstract

**Purpose:**

With the increasing use of MR‐guided radiation therapy (MRgRT), it becomes important to understand and explore accuracy of medical dosimeters in the presence of magnetic field. The purpose of this work is to characterize metal‐oxide‐semiconductor field‐effect transistors (MOSFETs) in MRgRT systems at 0.345 T magnetic field strength.

**Methods:**

A MOSFET dosimetry system, developed by Best Medical Canada for *in‐vivo* patient dosimetry, was used to study various commissioning tests performed on a MRgRT system, MRIdian^®^ Linac. We characterized the MOSFET dosimeter with different cable lengths by determining its calibration factor, monitor unit linearity, angular dependence, field size dependence, percentage depth dose (PDD) variation, output factor change, and intensity modulated radiation therapy quality assurance (IMRT QA) verification for several plans. MOSFET results were analyzed and compared with commissioning data and Monte Carlo calculations.

**Results:**

MOSFET measurements were not found to be affected by the presence of 0.345 T magnetic field. Calibration factors were similar for different cable length dosimeters either placed at the parallel or perpendicular direction to the magnetic field, with variations of less than 2%. The detector showed good linearity (R^2^ = 0.999) for 100–600 MUs range. Output factor measurements were consistent with ionization chamber data within 2.2%. MOSFET PDD measurements were found to be within 1% for 1–15 cm depth range in comparison to ionization chamber. MOSFET normalized angular response matched thermoluminescent detector (TLD) response within 5.5%. The IMRT QA verification data for the MRgRT linac showed that the percentage difference between ionization chamber and MOSFET was 0.91%, 2.05%, and 2.63%, respectively for liver, spine, and mediastinum.

**Conclusion:**

MOSFET dosimeters are not affected by the 0.345 T magnetic field in MRgRT system. They showed physics parameters and performance comparable to TLD and ionization chamber; thus, they constitute an alternative to TLD for real‐time *in‐vivo* dosimetry in MRgRT procedures.

## 1 INTRODUCTION

Magnetic resonance image‐guided radiation therapy (MRgRT) has gained impetus recently. ViewRay^®^ (ViewRay Inc., Oakwood, OH) is one of the first MRgRT treatment modalities with an onboard MR scanner attached to a teletherapy ^60^Co beam.^1^ The MRIdian^®^ Linac system which combines MR imaging and a 6 MV beam is the latest system developed by ViewRay. MRgRT offers many advantages including real‐time imaging, accuracy in treatment delivery, higher soft tissue contrast, and no additional imaging dose.^2^ Though there are significant advantages of these hybrid treatment systems with onboard MR scanners, these present a unique challenge to dosimetric measurements with some of the conventional radiation detectors, due to possible magnetic field susceptibility.

Several dosimeters are used for quality assurance in radiotherapy; these include ionization chambers (IC), thermoluminescent dosimeters (TLD), film, and active dosimeters such as diodes and metal‐oxide‐semiconductor field‐effect transistor (MOSFET).^3^, ^4^

For *in‐vivo* patient dose verification, the TLD, diodes, and MOSFETs are well proven for several radiotherapy beam modalities.^4^, ^5^, ^6^, ^7^ MOSFETs have matured to an extremely practical level of use in clinical settings. They offer a number of advantages including a small physical size, µm, thick active area, persistence of signal post‐irradiation, no dose rate and no temperature dependence, good linearity and isotropic response, and real‐time dose readouts.^5^, ^8^ Due to their small size, MOSFETs minimally perturb the radiation field, and are suitable for dosimetry of small fields and peripheral regions, steep dose gradients, and regions of electronic disequilibrium, like surfaces and tissue interfaces.^6^, ^9^ Instant readout and immediate re‐use allow subsequent measurements to be taken without removal of the detector; this makes the MOSFET a good alternative for the TLD.^6^

As drawbacks, MOSFETs show a time dependence of the readout (fading),^8^ especially for very small doses (<1 cGy), and a relatively short life, that is, maximum cumulated dose. It is also known that the magnetic field affects semiconductor devices^10^, ^11^ as in some conditions it deviates carriers toward the Si/SiO_2_ interface, resulting in change of the magnetoresistance of the conduction channel. This effect is known as the Hall effect, as a small electric field is generated as a result of a magnetic field applied perpendicular to the direction of the drain current, resulting in change of the space charge at the MOSFET conduction channel.^11^

In MRgRT, the electron return effect (ERE)^12^ is known to be significant in the presence of the magnetic field at phantom–air interface during irradiation, and this can influence dose measurements especially with IC.^13^ This effect is related to secondary electrons generated in the phantom media, which experience helical trajectories as they are subjected to the Lorentz force from the magnetic field when they exit the phantom; this results in re‐entry of these electrons to the phantom and hence dose increases at the phantom–air interface.^12^

Recently, preliminary studies were done on MOSFET detectors for use in MR‐IGRT ^60^Co based modality with a 0.345 T magnetic field.^14^, ^15^ The effect of magnetic field was investigated with regard to depth dose, linearity of response, and angular dependence. No significant difference in dose at depth was found with or without the magnetic field.^15^ Knutson et al.^14^ reported a slight increase in MOSFET response (~5%), attributed to induced currents from the dynamic magnetic field; hence, they advised to perform a special calibration procedure to account for the change in detector response to the dynamic magnetic field. According to Thorpe et al.,^16^ MOSFET‐based dosimeters, under the influence of 1 Tesla magnetic field, can be used as dosimeters for MRI‐guided radiotherapy (MRI Linac) with no effect of the magnetic field.

In this study, we intend to perform an extensive characterization of the commercially available MOSFET dosimeters (Best Medical Canada) in the MRIdian^®^ Linac system settings for several radiation characteristics and for typical patient MRgRT treatments, and compare them to TLD and IC commissioning results, to determine their suitability as an *in‐vivo* patient dose verification tool.

## 2 MATERIALS AND METHODS

The MOSFET dosimetry system (TN‐RD‐70W mobileMOSFET), developed by Best Medical Canada for *in‐vivo* patient dosimetry, was used for this study. The system is composed of a wireless battery operated reader, connected to five MOSFET dosimeters, allowing real‐time reading of dose in a single or sequential operation mode.

Two types of MOSFET dosimeters were used: TN‐502RD with cable length of 1.8 m (short) and TN‐502‐RD‐10 with cable length of 3.25 m (long). The long cable MOSFET is mostly used in this study, as it is deemed suitable for dosimetry, with less disturbance from the MR magnetic field to the MOSFET reader, as it extends beyond the 5 Gauss line. Comparison of response with the short MOSFET was done in the MR‐Linac setting. For all measurements, the standard sensitivity MOSFET (TN‐502RD), under standard bias sensitivity setting, was used.

The MOSFET detector has a sensitive area of 0.2 mm^2^ × 0.2 mm^2^ and 0.5 μm in SiO_2_ thickness^5^, ^6^. The silicon chip is packaged on a polyimide (Kapton) flexible circuit, sealed with an organic epoxy of 1.8 g/cm^3^ density, resulting in 1.3 mm total thickness and 2.5 mm width dosimeter. The MOSFET physical build‐up due to epoxy is approximately 0.8 mm, corresponding to an inherent water equivalent build‐up of ~1.5 mm, when corrected for density.

The MOSFET dosimeter mode of operation is described elsewhere.^6^, ^8^ Briefly, the detector is a dual P‐type MOSFET composed of two transistors, where hole transport dominates the channel current. The threshold voltage, V_th_, which is the gate voltage allowing current conduction through the drain to the source, changes with radiation dose; this parameter change, ∆V_th_ in mV, is proportional to the dose and is measured to establish the calibration factor of the dosimeter.

All MOSFET measurements were performed on MRIdian^®^ Linac system equipped with 6 MV flattening filter free (FFF) inline linac. Preliminary MOSFET measurements were performed with the 0.345 T magnetic field Off and then On for 100–600 MUs range and for a 10 cm^2^ × 10 cm^2^ equivalent field size, to check MOSFET immunity to the magnetic field. For all other measurements, the magnetic field was turned On. MRIdian^®^ Linac was commissioned to deliver 1 cGy/MU at d‐max, 90 cm source to axis distance (SAD) with 600 MU/min dose rate (DR). Measurements acquired with the MOSFET were compared with the commissioning data measured with Exradin ion chamber A28 with collecting volume of 0.125 cm^3^, waterproof edge diode detector from Sun Nuclear Corporation, and TLD‐100™ chips from Accredited Dosimetry Calibration Laboratory (ADCL). All measurements were done in solid water with MOSFET active volume facing the photon beam. Bolus sheet of 5 mm thickness was placed on the MOSFET dosimeter to prevent any air gap formation between the dosimeter and the phantom material. At least 5 minutes waiting time was allowed between two consecutive measurements for dosimeter stabilization and to avoid dose fading effect.

We characterized the MOSFET dosimeter with different cable lengths by determining its calibration factor, monitor unit (MU) linearity, angular dependence, field size dependence, percentage depth dose (PDD) variation, and output factor (OF) change. In addition, we performed intensity modulated radiation therapy quality assurance (IMRT QA) using MOSFETs and compared its results with IC measurements. Setup conditions for each test are described below.

### 2.1 Calibration

**Figure 1:**
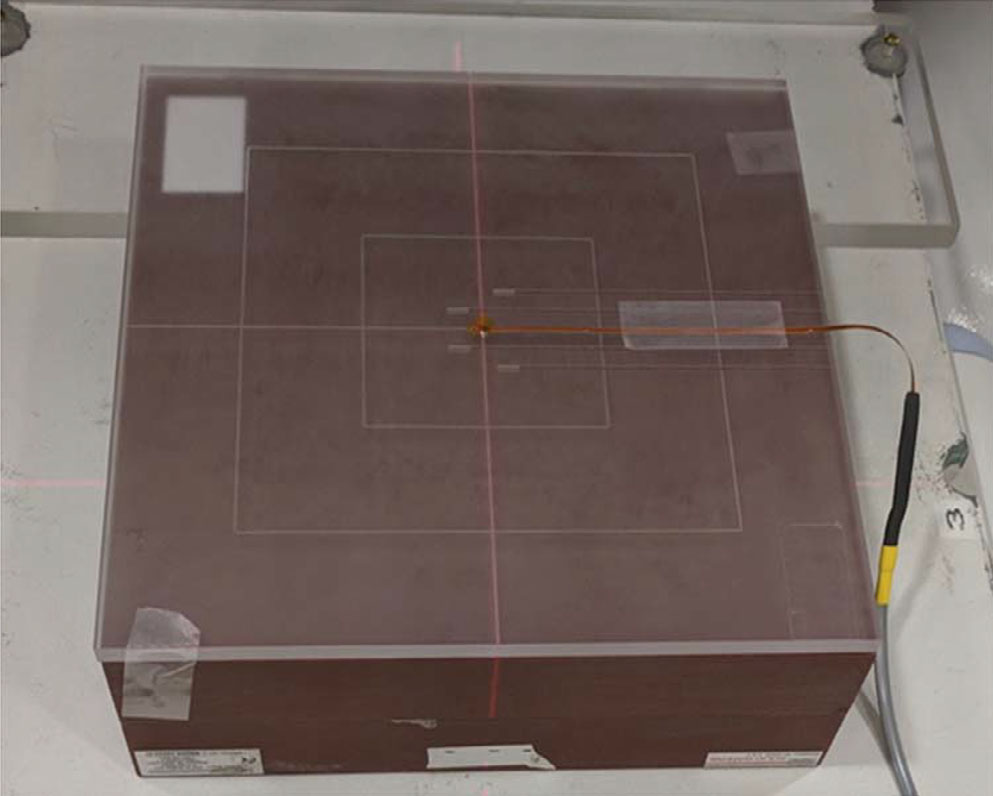
Setup used for metal‐oxide‐semiconductor field‐effect transistors (MOSFET) calibration: MOSFET was placed parallel and perpendicular to the magnetic field. MOSFET aligned perpendicular to the main magnetic field is shown in the figure.

The calibration factor is used to convert the measured detector response in mV to dose in cGy. Each MOSFET was calibrated before its use. MOSFETs of different lengths (long and short) were calibrated to evaluate the cable length effect on the calibration factor. Calibration was also performed by aligning the MOSFET parallel and perpendicular (Fig. 1) to the main magnetic field orientation.

At gantry 0° (beam vertical to dosimeter blob surface), 200 MUs were delivered at 1.5 cm depth, 90 cm (SAD), using 9.96 cm^2^ × 9.96 cm^2^ field size.

### 2.2 Monitor unit linearity

To determine the useful dynamic range of the MOSFET, 100–600 MUs were delivered in a step size of 100 MUs at 1.5 cm depth, 90 cm SAD, with 9.96 cm^2^ × 9.96 cm^2^ field size, and gantry parked at 0°. Three dose measurements for each MU setting were recorded, and the average MOSFET response in mV was determined.

### 2.3 Output factor

Field size dependence was studied from 0.83 to 20.75 cm^2^ field sizes at 5 cm depth, at gantry 0°. For each field size, dose was recorded for 200 MUs delivered at 90 cm SAD. Two dose measurements for each MU setting were recorded, and the average MOSFET response, normalized to the 9.96 cm^2^ × 9.96 cm^2^ field size, was used to determine the output factor. Results were compared to the IC data.

### 2.4 Directional dependence

**Figure 2:**
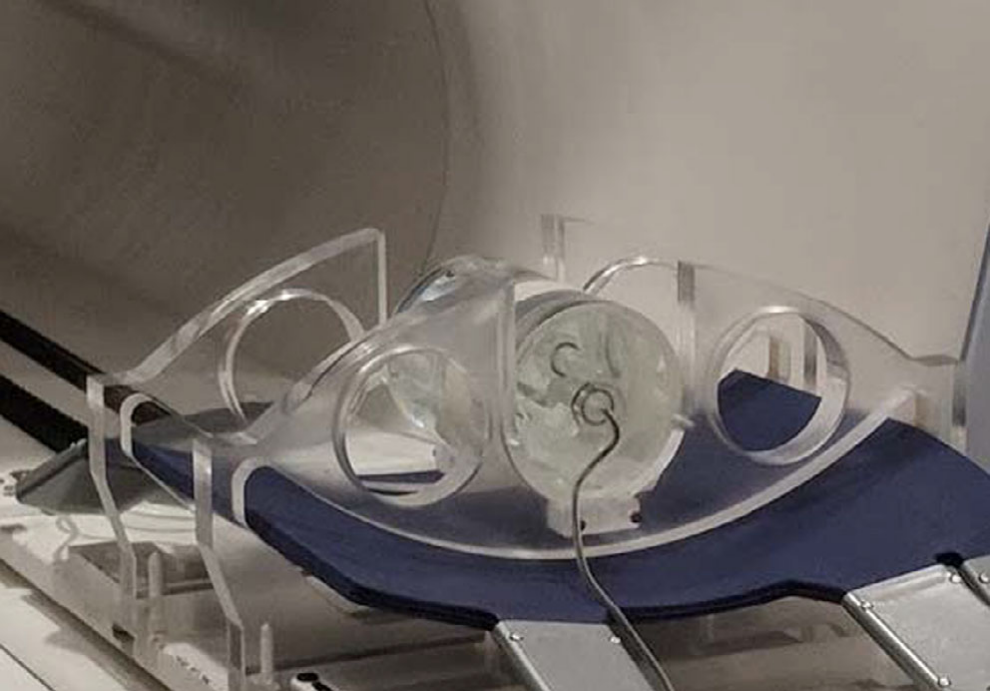
ViewRay QA cylindrical water phantom setup used to investigate directional dependence using ionization chamber and metal‐oxide‐semiconductor field‐effect transistors (MOSFET).

Directional dependence of MOSFET was investigated by placing the detector in a cylindrical water phantom (ViewRay Inc. Cleveland, OH). 200 MUs were delivered from different gantry angles: 0° to 330° range, with 30° increments, and for a field size of 9.96 cm^2^ × 9.96 cm^2^. Beams passing through the couch, with inherent attenuation, were included. Figure 2 shows the setup. TLD and IC measurements were performed in the same phantom setup and gantry rotations. MOSFET response was the average of three dose readings for each gantry angle. All measurements were normalized to the average response for all angles and for each detector. The setup used allows only relative comparisons of angular response between detectors and is anisotropic in nature; hence, quantitative angular dependence data cannot be inferred from these measurements.

### 2.5 Percentage depth dose and Monte Carlo simulations

The radiation energy spectrum changes with the depth of material it traverses, and a good detector should have minimum change in response at different depths. PDD measurements were performed for MOSFETs at 9.96 cm^2^ × 9.96 cm^2^ field size, 90 cm SAD, 200 MUs, and with 10 cm solid water backscatter. All measurements were performed in solid water for depths ranging from 0 to 15 cm for the MOSFET and from 1 to 15 cm for the IC. Two dose measurements for each depth were recorded for each detector, and the average response, normalized to the Dmax response, was used to determine the PDD curve.

The MRIdian^®^ Treatment Planning and Delivery Software (TPDS) enables the creation of treatment delivery and QA plans for the MRIdian^®^ system. The software uses Monte Carlo (MC) algorithm, with magnetic field component included, to track particle trajectories and dose deposition in phantom or patient media. The Advanced Dose Setting feature of the TPDS was used to set the grid resolution (1 mm), the number of histories (2 400 000) for the optimization, and the uncertainty value (0.2%). The MC algorithm was validated independently by Wang et al.^17^ for IMRT treatment verification in the presence of 0.35 T magnetic field.

Dose simulations were performed for water depths of 0.15, 0.6, 1, and 1.6 cm. The lowest depth (0.15 cm) was used to account for MOSFET inherent build‐up when placed at 0 cm depth. This build‐up was ignored for the other depth simulations. The 0.15 cm thin layer from the surface simulates skin thickness of clinical concern for skin toxicity in radiation therapy.

### 2.6 IMRT QA

**Figure 3:**
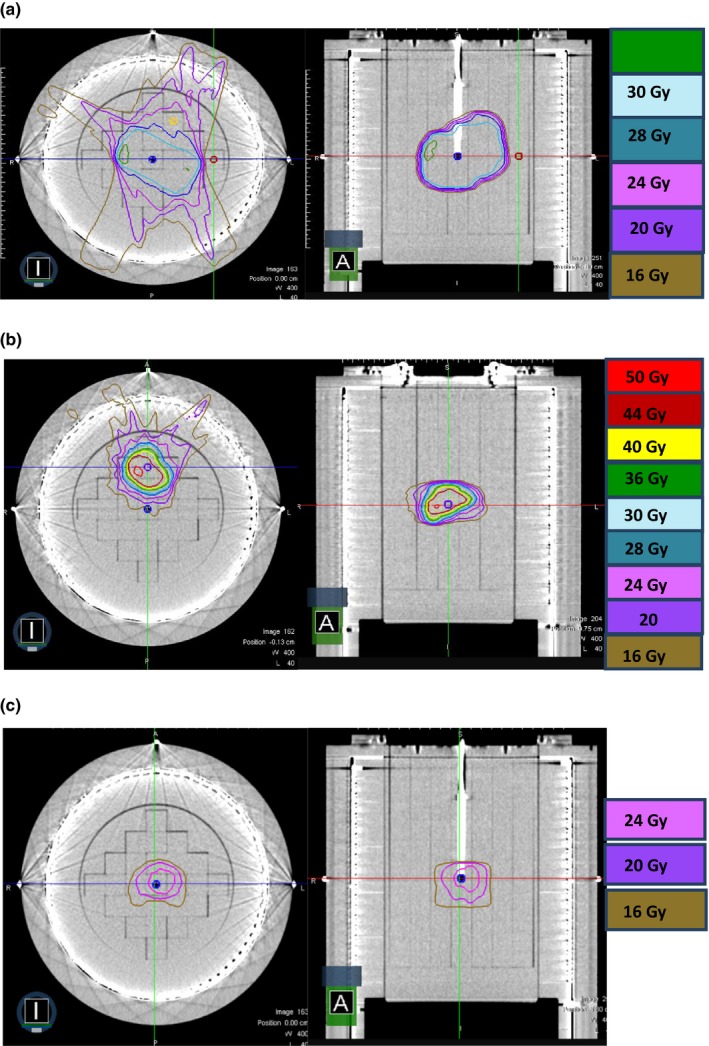
Radiation treatment plan dose calculated on MR compatible ArcCHECK for three different sites: (a) mediastinum, (b) liver, and (c) spine, respectively, planned for 30, 50, and 27 Gy. Point dose measurements were made using ionization chamber and metal‐oxide‐semiconductor field‐effect transistors (MOSFET).

**Figure 4:**
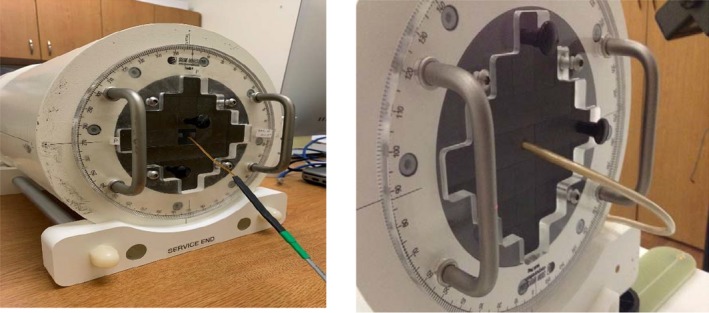
ARCCHECK setup used for intensity modulated radiation therapy quality assurance (IMRT QA) using ionization chamber (right) and metal‐oxide‐semiconductor field‐effect transistors (MOSFET) (left).

IMRT QA was performed for three clinical sites: mediastinum, liver, and spine. Liver was planned for 50 Gy in 5 factions, spine was planned for 24 Gy in single fraction, and mediastinum was a conventional treatment for 30 Gy in 10 fractions. QA plans were generated on MRIdian^®^ linac treatment planning system (TPS). MR compatible ArcCHECK^®^ (Sun Nuclear Corporation, Melbourne, FL, USA) was used for IMRT dose verification. A special holder was built to hold the MOSFET at the point of measurement. The active volume of the MOSFET was facing gantry 0° for all measurements. IMRT QA dose distribution is shown in Fig. 3. MOSFET measurements were compared to IC measurements. IMRT QA setup for treatment delivery is shown in Fig. 4.

## 3 RESULTS

### 3.1 Calibration

Calibration factors for MOSFET detectors with different cable lengths are shown in Table 1. Consistency in the MOSFET calibration factor over different cable lengths was observed. Due to an insignificant difference in calibration factors for different cable lengths, MOSFETs with longer cables were used in this study. The long cable MOSFET allowed safer distances between the dosimeters and the reader, placed outside the 5 Gauss line.

**Table 1:** Calibration factor for different MOSFET detector lengths.

MOSFET length	Delivered MUs	MOSFET response (mV)		Average (mV)	CF (mV/cGy)
Short	200	221.35	221.95	221.65	1.11
Short	200	221.45	222.15	221.8	1.11
Long	200	221.19	222.35	221.77	1.11
Long	200	221.26	222.25	221.76	1.11

MOSFET, metal‐oxide‐semiconductor field‐effect transistors.

Placement of the MOSFET detectors parallel or perpendicular to the magnetic field resulted in similar calibration factors, as shown in Table 2; the maximum percentage difference in calibration factor for the two setups was 1.8%. This number is within the detector reproducibility of 2.4% at two standard deviations (SD) as shown in Table 2. Chuang et al.^6^ reported a reproducibility measurement of 1.5% at 1 SD for 1 Gy at 6 MV beam energy. For all measurements, calibration factors were within the range of 1.11–1.14 mV/cGy for all detectors. Kinhikar et al.^18^ reported calibration factors within 1.10–1.12 mV/cGy and 2% SD for a 6‐MV photon beam with a helical tomotherapy. The results obtained in the MRgRT linac setting are within the range of values reported elsewhere for conventional radiotherapy linacs.^6^, ^18^

**Table 2:** MOSFET response when aligned parallel and perpendicular to magnetic field orientation.

Dose (MUs)	Parallel to magnetic field		Perpendicular to magnetic field	
	MOSFET (mV)	*CF (mV/cGy)	MOSFET (mV)	*CF (mV/cGy)
200	227.64	1.14	227.52	1.14
200	222.28	1.11	225.56	1.13
200	222.4	1.11	225.56	1.13
200	222.45	1.11	229.51	1.15
		1.12 Average		1.14 Average
		1.18 SD (%)		0.83 SD (%)

MOSFET, metal‐oxide‐semiconductor field‐effect transistors.

* Calibration factor.

The MOSFET measurements with the MR magnetic field turned On, in comparison to the Off condition, did not influence the MOSFET dose response; the dose response ratio was within ±2% for 100–600 MUs dose range, which is within the dosimeter reproducibility. The 0.345 T magnetic field of the MRIdian^®^ linac does not seem to affect MOSFET measurements, in agreement with results obtained by Lysenko et al.^10^ for P‐type MOSFET threshold voltage, for magnetic fields up to 1.5 Tesla, and with no source–substrate bias. Thorpe et al.^16^ reported no significant change in the MOSFET current voltage characteristics, I (V), with a magnetic field of 1 T, turned On or Off, at different setup configurations; hence, the MOSFET threshold voltage was deemed not affected by the magnetic field.

### 3.2 Linearity

**Figure 5:**
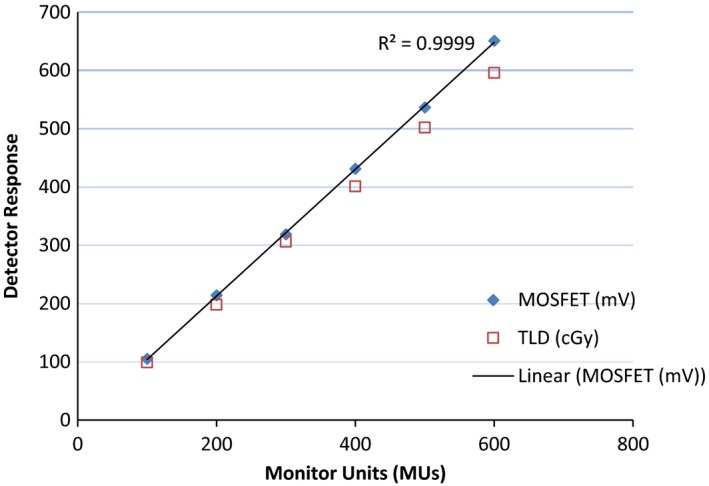
Monitor unit linearity results for metal‐oxide‐semiconductor field‐effect transistors (MOSFET) and thermoluminescent detector (TLD).

MU linearity test results for the MOSFET and TLD are shown in Fig. 5. The MOSFET showed a linear response for 100–600 MUs doses, with a linear fit error R^2^ = 0.999. This is consistent with several findings^5^, ^6^ reported for external beams with no magnetic fields. Chuang et al.^6^ reported a linear response with a correlation coefficient R^2^ of 0.998 from 5 to 420 cGy for 6 MV IMRT beam. Ramaseshan et al.^5^ reported a true linear response at 6 MV photons for 5–500 cGy dose range, with a correlation coefficient R^2^ of 0.999.

### 3.3 Output factor

**Figure 6:**
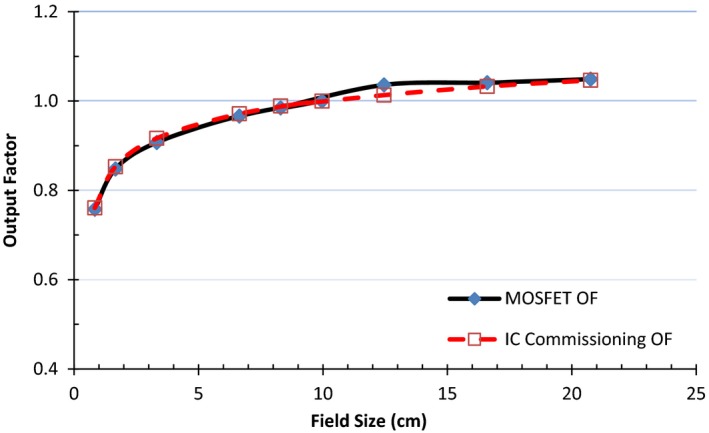
Output factor for various field sizes for metal‐oxide‐semiconductor field‐effect transistors (MOSFET) compared to ionization chamber.

The output factor for MOSFET and IC is shown in Fig. 6. The MOSFET behavior is consistent with the IC results. The absolute discrepancy in output factors for the two detectors was within 0.2%–2.2% for all field sizes investigated. This difference is close to the 2% reproducibility of the dosimeter. The average MOSFET output factor for 0.83 to 20.75 cm field sizes was 0.954; the same as the IC. The estimates of the output factor standard deviation were 10.4% and 9.9% for MOSFET and IC, respectively, for the fields of interest. The consistency of the MOSFET output factor and its small size make it an attractive alternative to the ionization chamber to verify the output factor at different field sizes.

### 3.4 Directional dependence

**Figure 7:**
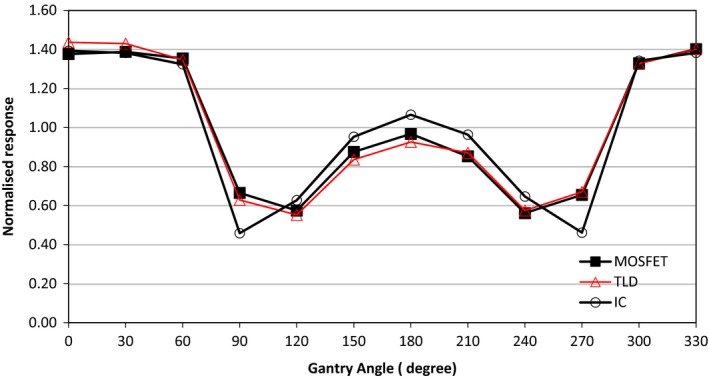
Directional dependence results using cylindrical water phantom for metal‐oxide‐semiconductor field‐effect transistors (MOSFET), thermoluminescent detector (TLD), and ionization chamber measured for gantry angles 0° to 330°. Detector normalized response is plotted as a function of gantry angle.

Figure 7 shows the directional dependence results for MOSFET, TLD, and IC measured in ViewRay cylindrical phantom. The maximum percent difference of 5.5% between MOSFET and TLD was observed for 90°; for all other angles, this difference varied between 0% and 4.5%. MOSFET and TLD showed discrepancies with IC measurements of 31% at 90° and 29.6% at 270°. Normalized responses for all detectors showed strong anisotropy for gantry rotations at angles between 90° and 270° in comparison to 0° to 60° rotation; this systematic behavior is to be related to the phantom setup, likely not suitable for absolute detector angular dependence measurement, and to the table couch material attenuation. It should be noted that MOSFET angular dependence within 2.5% was reported for 0° to 180° angle rotation for external beam radiotherapy, where MOSFET was inserted inside a cylindrical phantom.^6^

### 3.5 Percentage depth dose and Monte Carlo simulations

**Figure 8:**
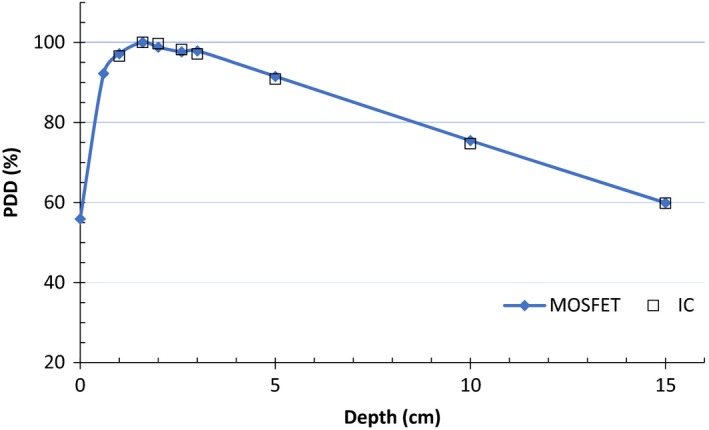
Percentage depth dose (PDD) measurements with metal‐oxide‐semiconductor field‐effect transistors (MOSFET) and ionization chamber for depths from 0 to 15 cm.

PDD measurements with MOSFET and IC are shown in Fig. 8 for depths from 0 to 15 cm. The MOSFET data show agreement with IC measurements, with maximum discrepancy of 1% for 10 cm depth. These results agree with PDD measurements for MOSFETs reported by Chuang et al.^6^ for 6 MV IMRT beams, where discrepancies of less than 3% were found between MOSFET and IC data for depths ranging from 0.5 to 34 cm.

MOSFET measurements were also performed at regions of zero or partial build‐up (0 and 0.6 cm) before Dmax, thanks to MOSFET's small size which allows measurements at regions of lack of electronic equilibrium. The comparison of these PDD measurements with MC calculations showed agreement within 5% for depths ranging from 0.15 to 1.6 cm. The 5% maximum discrepancy was observed at the MOSFET surface measurement (0.15 cm) where PDDs were 55.9% and 53.3% for MOSFET measurement and MC calculation, respectively. In another study by Xiang et al.^19^ on MOSFET PDD response compared to MC calculations in the build‐up region (0.1 to 2 cm), done at 6‐MV energy and with no magnetic field, an agreement within 3%–5% was found, similar to our result (5%). The study also showed that MOSFET PDD measurements were 60.3% and 42% at 2 and 1 mm depths, respectively.^19^ It should be noted that the measured 55.9% MOSFET PDD in this study, at 1.5 mm equivalent depth, is within the reported values by Xiang et al.^19^ for similar depth range; this makes the MOSFET dose measurement at the surface not affected by the lower magnetic field of 0.345 T.

### 3.6 IMRT QA

IMRT QA plan for mediastinum, liver, and spine are shown in Fig. 3(a‐c). IMRT QA for these plans was performed using the MOSFET dosimeter and compared to IC results. Results of IMRT QA are summarized in Table 3. The percentage difference between IC and MOSFET was 0.91% for liver, 2.05% for spine SBRT, and 2.63% for mediastinum. Maximum difference between planned and MOSFET results was noticed for spine due to longer treatment time. This error is within the 5% accepted clinical value for conventional IMRT 6 MV beams; in particular, for *in‐vivo* dosimetry in patients treated for head and neck cancer, it has been shown that the MOSFET measured dose and the dose calculated by the planning system agreed within 5%.^6^, ^9^, ^20^, ^21^ These results make MOSFET attractive for *in‐vivo* dose verification during IMRT QA in MRgRT at 0.345 T magnetic field strength.

**Table 3:** IMRT QA results for clinical sites: mediastinum, liver, and spine. Comparison between dose planned and measured with MOSFET and IC is listed below.

Anatomical site	Target volume (cc)	Dose/fraction (cGy)	Number of fractions	Planned dose (cGy)	Average MOSFET reading (cGy)	IC reading (cGy)	Planned‐MOSFET difference (%)	Planned‐IC difference (%)	IC‐MOSFET difference (%)
Mediastinum	58.27	3	10	269.5	262.5	269.6	−2.6	0.04	2.63
Liver	33.6	10	5	950.2	960.5	969.3	1.08	2.01	0.91
Spine	17.2	27	1	2496	2375	2424.7	−4.85	−2.86	2.05

IC, ionization chamber; IMRT QA, intensity modulated radiation therapy quality assurance; MOSFET, metal‐oxide‐semiconductor field‐effect transistors.

## 4 DISCUSSION

*In‐vivo* dosimetry is an important part of patient quality assurance (QA) in radiotherapy. ViewRay's MRIdian^®^ Linac system is relatively a new treatment modality with fully integrated MR‐guided, 6 MV FFF photon beam. There is no standardized report for guidelines on QA devices and procedures for MRgRT, and therefore it is critical to perform careful characterization of dosimeters before use in clinical practice.

MOSFETs were clinically proven dosimeters for quality assurance of conventional IMRT beams with no magnetic fields^5^, ^6^, ^9^, ^20^ as they are small in size, energy independent, linear, and isotropic, hence allowing dose measurements in high dose gradient fields at multiple energies and beam orientations.

It was our intent to extend their use for MRgRT modality as an *in‐vivo* dosimeter; to this end, extensive characterization of its radiation characteristics in the presence of the low magnetic field of 0.345 T, used by the ViewRay's MRIdian^®^ Linac system, was performed.

The magnetic field is known to affect semiconductor devices^10^, ^11^ as in some conditions it deviates carriers toward the Si/SiO_2_ interface, resulting in change of the magnetoresistance of the conduction channel. In our MOSFET measurements, the effect of the magnetic field on detector response was found to be negligible and within the detector reproducibility; this is likely related to the detector operation in the low drain current region at the low onset V_th_ voltage, which makes the channel magnetoresistance and Hall effect negligible.^10^

In the presence of the magnetic field and with no air gaps, the ERE effect^12^ is unlikely to affect the MOSFET readouts especially when the dosimeter is placed under full build‐up material at Dmax (~1.5 cm), as described in the above calibration test setup. This is consistent with the observed MOSFET behavior during calibration, with MR magnetic field turned On or Off.

In the special case where MOSFETs are used with partial or no build‐up, such as in surface dose measurements, the ERE effect was found in this study to have no effect on dose measurements, contrary to what was anticipated for phantom–air interface at higher magnetic fields.^12^, ^13^ Indeed, Raajmakers et al.^12^ estimated the mean gyroradius of electrons returning to a phantom–air interface to be 2.2 mm at 1.5 T; this short range of electrons was anticipated to result in significant dose deposition to the dosimeter at higher magnetic field. In the MRIdian^®^ Linac of 0.345 T magnetic field strength (~quarter of 1.5 T), the mean gyroradius, which is inversely proportional to the magnetic field,^13^ will be roughly ~9 mm at 0.345 T. At this range, and given the small dimensions of the MOSFET dosimeter (1.3 mm), the secondary electrons exiting the surface–air interface will likely scatter or recombine in air, with negligible ERE contribution to the dosimeter.

Good agreement of the PDD results for MOSFET and IC commissioning data shows that MOSFET can be used clinically to determine the dose at a point beyond Dmax during radiotherapy treatment verification on the ViewRay MRIdian^®^ system. PDD data in the build‐up region compared to MC simulations showed that MOSFETs are suitable for *in‐vivo* dose verification in MRgRT setting, especially for surface dose measurements.

The cable length of the MOSFET (1.8 vs. 3.25 m) did not show significant effect of the magnetic field on the MOSFET response and its calibration factor. To prevent any electromagnetic disturbance on the reader electronics due to the magnetic field, it is advised to use the long cable MOSFET for routine *in‐vivo* dosimetry, as it allows the reader to be located outside the 5 Gauss line limit. No effect of the detector orientation with respect to the magnetic field direction was observed; this makes the detector placement on the surface flexible for all orientations.

Output factors are an inherent characteristic of an accelerator and are measured periodically to ensure the proper functioning of the linac. MOSFET measurements performed for field size ranging from 0.83 to 20.75 cm^2^ showed good agreement with the commissioning data; this shows that the MOSFET can be a practical secondary detector for this measurement for small field sizes.

Angular dependence measurements of MOSFETs in MRgRT setting showed discrepancies with measurements reported in standard IMRT beam radiotherapy settings.^6^ MOSFET and TLD showed agreement within 5.5% for all angle measurements. It is not clear why IC, MOSFET and TLD showed strong attenuation of response at angles of 90° and 270° and why IC measurements showed discrepancies up to 31% at these angles with both MOSFET and TLD dosimeters. It is important to carefully select the phantom measurement setup during dose measurements for beam orientations transverse to the couch table to account for beam attenuation.

In this study, we demonstrated the use of MOSFET for IMRT QA for different anatomical sites in MRIdian^®^ linac setting. Overall, MOSFET results showed small discrepancy with TPS planned dose values, within 5% error for all sites. Amin et al.^9^ reported agreement between MOSFET measurements and calculated doses by the treatment planning system for head and neck and prostate IMRT plans within 0.47 ± 2.45% for conventional beams. In another study,^19^ overall MOSFET error was reported to be composed of several errors, including detector reproducibility (2%), angular dependence (3%), and systematic error due to phantom setup (2%), which when combined could play an important role in dose measurement accuracy. Overall, the error in this study is within the 5% accepted clinical value for routine IMRT 6 MV beams^9^; we conclude that MOSFETs can be used for standard dose fraction IMRT QA as well as for a single fraction SBRT treatment for MRgRT modality at 0.345 T magnetic field strength.

There are a few drawbacks of the MOSFET detector. It has limited lifespan, requires connection to bias voltage during irradiation, and requires longer cables in MR suite. Also, current design of MOSFET is not capable of providing sufficient signal for MR imaging. We believe that the standard sensitivity microMOSFET (TN‐502RDM),^5^ with smaller width of 1 mm (2.5 mm for the standard MOSFET) with addition of MR compatible marker, will significantly improve the MR image quality. This will help to establish the relation between internal organ motion and skin surface for treating patients on a non‐gated system.

Nevertheless, MOSFET physics characteristics in MRgRT at 0.345 T are shown in this study to be similar to the parameters measured for conventional IMRT beams. MOSFET inherent advantages, such as their simple setup, compact size and instantaneous readouts, online dosimetry, and maintenance of adequate linearity over its lifespan, make it a suitable dosimetry tool for *in‐vivo* IMRT QA for MRgRT beams at 0.345 T magnetic field strength.

## 5 CONCLUSION

MOSFET dosimeter characterization parameters in the presence of 0.345 T magnetic field were validated. They showed performance comparable to TLD and IC within acceptable error in 6 MV FFF MRIdian^®^ Linac used in low magnetic field of MRgRT treatment modalities. Results show that MOSFETs are a viable alternative to TLD for real‐time *in‐vivo* dosimetry, and can be used successfully for major beam characterization tests for MRgRT procedures. Minor limitations were identified.

## CONFLICT OF INTEREST

The authors declare no conflict of interest.
